# General Practitioner’s Practice in Romanian Children with Streptococcal Pharyngitis

**DOI:** 10.3390/medicina61081408

**Published:** 2025-08-02

**Authors:** Reka Borka Balas, Lorena Elena Meliț, Ancuța Lupu, Boglarka Sandor, Anna Borka Balas, Cristina Oana Mărginean

**Affiliations:** 1 Department of Pediatrics I, “George Emil Palade” University of Medicine, Pharmacy, Sciences and Technology of Targu Mures, Gheorghe Marinescu Street, No. 38, 540136 Targu Mures, Romania; rekaborkabalas@gmail.com (R.B.B.); marginean.oana@gmail.com (C.O.M.); 2 Department of Pediatrics II, “George Emil Palade” University of Medicine, Pharmacy, Sciences and Technology of Targu Mures, Gheorghe Marinescu Street, No. 38, 540136 Targu Mures, Romania; 3 Department of Pediatrics, University of Medicine and Pharmacy Grigore T. Popa Iasi, Universității Street No. 16, 700115 Iasi, Romania; anca_ign@yahoo.com; 4 “George Emil Palade” University of Medicine, Pharmacy, Sciences and Technology of Targu Mures, Gheorghe Marinescu Street, No. 38, 540136 Targu Mures, Romania; sandorbogi99@gmail.com (B.S.); annaborkabalas2003@icloud.com (A.B.B.)

**Keywords:** beta-hemolytic group A streptococcus, pharyngitis, children, general practitioner

## Abstract

*Background and Objectives*: A correct diagnosis of *beta-hemolytic group A streptococcus* (GAS)-pharyngitis allows the prevention of complications and unnecessary use of antibiotics. The aim of this study was to assess the management of pediatric GAS-pharyngitis in Romanian general practitioners (GPs)’ practice. *Material and Methods*: a cross-sectional study was conducted using a questionnaire distributed to Romanian GPs. *Results*: In total, 56 GPs completed the questionnaire, mostly females (83.9%, *n* = 47) from an urban area (60.7%, *n* = 34). They treated 5–10 (35.7%) or more than 10 (32.1%) cases of GAS monthly and considered white exudate on tonsils (92.9%, *n* = 52) to be the most suggestive clinical sign. Of the GPs, 25% (*n* = 14) used the Centor Criteria, 10.7% (*n* = 6) performed a rapid antigen detection test, and 42.9% (*n* = 24) requested throat culture for diagnosis. The younger GPs used the Centor Criteria significantly more often (*p* = 0.027) than the older ones. Most GPs (69.6%, *n* = 39) preferred targeted antibiotic therapy. Amoxicillin-clavulanate was the most commonly used antibiotic (55.4%, *n* = 31). Most GPs preferred oral antibiotics (89%, *n* = 50) for 10 days (55.4%, *n* = 31). *Conclusions*: Antibiotic treatment was initiated mostly based on clinical symptoms and in a short-course therapy. GPs stated that they prefer targeted antibiotic therapy, but they did not use proper diagnostic tools.

## 1. Introduction

Sore throat is one of the most common reasons for presenting to the general practitioner (GP), pediatrician or ear–nose–throat (ENT) specialists. Therefore, this symptom is most often treated in ambulatory setting and is definitely the chief symptom of pharyngitis. It is true that viruses are the most frequent etiology of pharyngitis, but beta-hemolytic group A streptococcus (GAS), also known as *Streptococcus pyogenes*, tops the list when it comes to bacterial causes. Although not so common as viral pharyngitis, according to the previous reports, approximately 600 million of new pediatric cases of symptomatic GAS-pharyngitis are diagnosed every year worldwide. Moreover, approximately 500,000 of them develop rheumatic fever, and in 300,000 cases, GAS-pharyngitis is complicated by rhematic carditis, especially in less developed and developing countries, where the prevalence is approximately 3 times higher when compared to developed countries [1]. According to WHO 2024 guidelines, the incidence of GAS-pharyngitis in the 5–14 age group is 22.1 episodes per 100 child-years, corresponding to 288.6 million episodes per year [2].

Taking into account the broad spectrum of severe complications related to GAS-pharyngitis, such as rheumatic fever or cardiac complication, it is extremely important to diagnose timely and precisely the presence of GAS infection. The diagnosis should be suspected in the presence of clinical symptoms and confirmed by a laboratory test. Several clinical scoring systems were developed for orienting physicians in the decision to test the patient for GAS-pharyngitis. Thus, the most commonly used system is the Centor Criteria, which includes four clinical criteria: fever >38 °C, edema or exudate of the tonsils, enlarged or painful anterior cervical lymph nodes in the lack of cough [3]. The modified McIsaac score added the age in The Centor Criteria stating that GAS-pharyngitis most commonly affects children between 3 and 14 years, being rare under the age of 3 years [4]. These clinical scoring systems are extremely useful especially in low-income settings, but the diagnosis of GAS-pharyngitis should be confirmed by a laboratory method, when possible, except when incontestable viral signs are present [5].

The laboratory tests that confirm the diagnosis of GAS-pharyngitis involve culture of the tonsils, which in fact is defined as the gold standard test and it can be achieved by two methods, i.e., rapid antigen detection test (RADT) and molecular test. The sensitivity of the RADT varies between 85 and 86%, while the specificity is even higher, between 95 and 96% [6,7]. The recommendations of the worldwide guidelines differ among geographical areas. The North America, France, and Finland guidelines and those from Israeli sustain the need for RADT or culture for the diagnosis of GAS-pharyngitis, and if positive, they strongly recommend antibiotic treatment. Contrariwise, the guidelines from England, Scotland, Belgium, and Netherlands consider that culture and antibiotic treatment are not needed since sore throat, most commonly caused by viruses, is usually a self-limiting disease [8]. Nevertheless, the proper diagnosis of GAS-pharyngitis might be important due to the fact that it not only reduces the unnecessary antibiotic prescription, but it is also the best prophylactic strategy for the occurrence of rheumatic fever and GAS-pharyngitis-related suppurative complications [9,10].

The emergence of multi-drug-resistant bacteria has become a major public health problem worldwide, especially in pediatric patients due to the trend to prescribe antibiotics even if not justified [11]. Nevertheless, it is true that antibiotics represent the first line treatment for GAS-pharyngitis, but the decision to recommend antibiotics should be individually tailored to the patient’s symptoms and evolution. Despite the contradictory evidence from the literature, the general practitioner (GP) and/or the pediatrician should judiciously choose between treating and not treating GAS-pharyngitis according to the patient’s complaints, comorbidities and his further risk to develop GAS-complications. The irrational use of antibiotics has led to critical problems in terms of drug-resistant bacteria emergences especially in developing countries [12,13]. Recent reports from a poor-income area proved an extremely high resistance rate of bacterial isolated from the throat swabs to ampicillin, which in fact is commonly used in these areas [13]. Still, the prevalence of invasive GAS disease is increased worldwide accounting for 663,000 new cases annually resulting in more than 150,000 deaths [1] raising once more the dilemma ‘To be or not to be?’—‘To treat or not to treat GAS-pharyngitis?’.

The aim of our study was to assess diagnostic and treatment patterns used by Romanian GPs in management of GAS-pharyngitis in children.

## 2. Materials and Methods

### 2.1. Participants

We randomly included GPs from two cities in the central area of Romania with over 50 children registered in their practice. Of the 110 GPs selected based on this criterion, only 56 responded to our questionnaire (50.9%).

### 2.2. Procedure

The cross-sectional study was conducted as an anonymous survey performed from January 2024 till April 2024 using a pilot questionnaire with 20 questions (File S1) distributed to GPs from Targu Mures, Odorheiu Secuiesc, and the surrounding villages of these two cities belonging to the central region of Romania.

The study was approved by the Ethics Committee of The Emergency Clinical County Hospital of Targu Mures, 29458/13.11.2023. All the data were only used for research and collected on a strictly voluntary and anonymous basis. All the participants provided informed consent.

The questionnaire assessed general sociodemographic variables such as age and gender; but also targeted questions about the number of children registered in their practice; the number of GAS-pharyngitis treated/per month in the last year; what symptoms are thought to be associated with GAS-pharyngitis; if they used the Centor Criteria for the diagnosis; whether a rapid antigen detection test (RADT) is used in their daily practice; do they request throat culture in all cases when GAS-pharyngitis is suspected; for which diseases do they perform differential diagnosis; do they start treatment after they have confirmed that the patient has GAS-pharyngitis; how do they treat GAS-pharyngitis and for how long; which antibiotic do they prefer; if access to medicines influenced their antibiotic choice; which route of antibiotic administration did they recommend; did they consider follow-up visit necessary; which are the indications for referring the patient to a pediatric specialist; did they use the ASLO titer to follow up patients and if they treated asymptomatic patients with high ASLO titers.

The relatively low response rate was due to certain objective reasons: the small number of children above the age of 3 years registered in the practice, lack of experience in this field, or simply time management. The study has a regional scope being limited to the experience of GPs from the central region of Romania.

### 2.3. Statistical Analysis

The statistical analysis included both descriptive (frequency, percentage), and inferential statistics. The Chi square test was applied to assess the potential correlations between the qualitative variables. The significance threshold chosen for the *p* value was 0.05. The statistical analysis was performed using the GraphPad software.

## 3. Results

### 3.1. Demographic Data and Practice

The final analyzed sample consisted of 56 GPs, among which 83.9% (*n* = 47) females and 16.1% (*n* = 9) males. The analysis of respondents’ age distribution of respondents was revealed that 39.3% (*n* = 22) were aged between 36 and 45 years, 21.4% (*n* = 12) were 56–65 years, 19.6% (*n* = 11) between 29 and 35 years, 12.5% (*n* = 7) were above 65 years, and 7.1% (*n* = 4) were aged between 46 and 55 years. We further divided the GPs into two age groups, the younger group was 29–45 years old (58.9%, *n* = 33), and the older age group included GPs above the age of 46 years (41.07%, *n* = 23).

In terms of practice, 60.7% (*n* = 34) of the GPs worked in urban areas, 39.3% (*n* = 22) in rural areas, and 66.1% (*n* = 37) had more than 200 children registered in their practice.

When asked about the number of children treated for GAS-pharyngitis in 2023, 35.7% (*n* = 20) of them treated 5–10 cases per month, 32.1% (*n* = 18) more than 10 cases, and 32.1% (*n* = 18) less than 5 cases per month (Table 1).

### 3.2. Clinical Signs and Symptoms

The association between swollen tonsils and white oropharyngeal exudates was considered the most common symptom of GAS-pharyngitis, reported by 92.9% (*n* = 52). The second most common sign was fever >38 °C (85.7%, *n* = 48), followed by swollen and tender anterior cervical adenopathy (82.1%, *n* = 46), while sore throat (73.2%, *n* = 41), and lack of appetite (42.9%, *n* = 24) were less frequently considered as suggestive symptoms.

### 3.3. Laboratory Tests

Only 25% (*n* = 14) of GPs used the Centor Criteria to diagnose GAS-pharyngitis and only 10.7% (*n* = 6) of them performed a RADT in their practice for suspected GAS-pharyngitis.

Nearly half (42.9%, *n* = 24) of GPs requested throat culture for the diagnosis of GAS-pharyngitis, but 57.1% (*n* = 32) considered throat culture unnecessary for the diagnosis. There was no statistically significant difference in the use of diagnostic tests between either GPs in urban and rural area (Centor Criteria *p* = 0.528, RADT *p* = 0.67, throat culture *p* = 0.968), or between men and women (Centor Criteria *p* = 0.423, RADT *p* = 1, throat culture *p* = 0.475).

Furthermore, we assessed the differences between age groups regarding the frequency of the preferred diagnostic methods, and we noticed that the younger age group (29–45 years) used the Centor Criteria significantly more often (*p* = 0.027) than the older age group (above 46 years of age). Nevertheless, no significant difference was found between the younger and older age groups for throat culture (*p* = 0.937), or RADT (*p* = 0.681).

In the case of patients presenting with swollen tonsils with white exudate, 19.64% (*n* = 11) of GPs used the Centor Criteria, 7.14% (*n* = 4) used RADT and 39.28% (*n* = 22) required throat culture.

### 3.4. Differential Diagnosis

Regarding the differential diagnosis of GAS-pharyngitis, GPs most often mentioned infectious mononucleosis (91.1%, *n* = 51) and viral pharyngitis (80.4%, *n* = 45), but 58.9% (*n* = 33) also considered scarlet fever, 64.3% (*n* = 36) rhinopharyngitis and 57.1% (*n* = 32) peritonsillar abscess as possible diagnosis.

### 3.5. Treatment of GAS Pharyngitis

When asked “Do you start treatment after you have confirmed clinically that the patient has GAS-pharyngitis”, 85% (*n* = 48) answered that they would start treatment immediately, while 15% (*n* = 8) only after they had a positive throat culture result.

Regarding the treatment method, most GPs (69.6%, *n* = 39) would use targeted antibiotic therapy. Nonetheless, 37.5% (*n* = 21) would start with empirical antibiotic therapy, 14.3% (*n* = 8) would prescribe symptomatic medication, and 7.1% (*n* = 4) would initiate only symptomatic treatment. Younger GPs (29–45 years) used targeted antibiotics more frequently (67%, *n* = 22) as compared to the older ones (33%, *n* = 8), but the difference was not statistically significant (*p* = 0.264).

**Figure 1:**
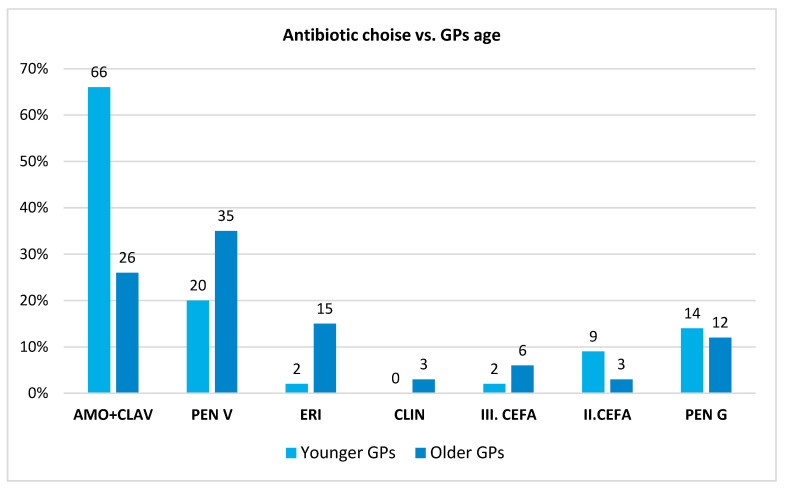
Antibiotic choice depending on the GP’s age. Legend: AMO + CLAV: Amoxicillin clavulanic acid; PEN V: Penicillin V; ERI: Erythromycin; CLIN: Clindamycin; III.CEFA: 3rd generation Cephalosporin; II.CEFA: 2nd generation Cephalosporin; PEN G: Penicillin G.

Concerning antibiotics, amoxicillin-clavulanate was the overall preferred antibiotic (55.4%, *n* = 31), followed by orally administered penicillin V (39.3%, *n* = 22) and intravenous penicillin G (17.9%, *n* = 10). However, a small number of GPs used 2nd or 3rd generation cephalosporins most often for GAS-pharyngitis (5%, *n* = 3 and 2%, *n* = 1, respectively). When assessing the preferred antibiotic depending on the GPs’ age, we observed that younger GPs preferred amoxicillin clavulanate (66%, *n* = 22/33 vs. 26%, *n* = 6/23), while older GPs started with Penicillin V more often (35%, *n* = 8/23 vs. 20%, *n* = 7/33), but the difference was not statistically significant (*p* = 0.563). In terms of intravenous treatment, younger GPs preferred Penicillin G and 2nd generation cephalosporins (Figure 1).

When asked which antibiotic they prefer as empirical therapy, 45% (*n* = 25) of GPs stated that they would start empirical antibiotic therapy with amoxicillin-calvulanate, 24% (*n* = 13) with penicillin V, 7% (*n* = 4) with erythromycin, 7% (*n* = 4) with 3rd generation cephalosporin, 14% (*n* = 8) with penicillin G, and 3% (*n* = 2) with 2nd generation cephalosporin.

In terms of route of administration, 89% (*n* = 50) of GPs would recommend oral treatment and only 9% (*n* = 5) would use intravenous antibiotics. There was a small number of respondents (2%, *n* = 1) who would start initially with intravenous and continue with orally administered antibiotics.

**Figure 2:**
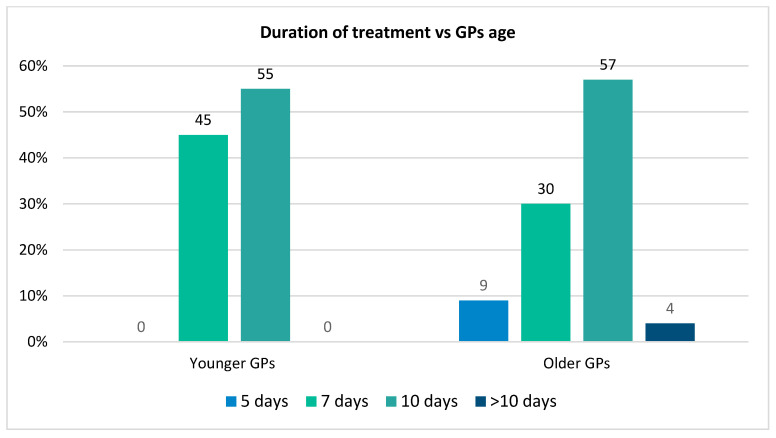
Duration of treatment depending on GPs age.

Nearly a half of GPs stated that they commonly prescribe a 10 day-antibiotics regimen (55.4%, *n* = 31), 39.3% (*n* = 22) used a 7 day-regimen, while 2 of them treated patients with GAS-pharyngitis for only 5 days (3.6%, *n* = 2), and 1 GP for more than 10 days (1.8%, *n* = 1). Younger GPs are more likely to treat GAS for only 7 days (45.45%, *n* = 15/33 vs. 30.43%, *n* = 7/23), but there is no significant difference in terms of 7 days (*p* = 0.07) or 10 days regimens (*p* = 0.883) between younger and older GPs (Figure 2).

As certain antibiotics are temporarily unavailable in Romania, we asked whether antibiotic choice is influenced by antibiotic availability and found that 89% (*n* = 50) of GPs took into account the availability of antibiotics at the time of prescription.

### 3.6. Follow-Up of a Patient with GAS Pharyngitis

After treatment is completed, 96.4% (*n* = 54) of GPs considered that a follow-up is needed for patients with GAS pharyngitis. When asked if they use anti-streptolysin O titer (ASLO) to follow up patients, 48.2% (*n* = 27) of GPs answered affirmatively, but 51.8% (*n* = 29) of them declared that they did not treat asymptomatic patients with an increased ASLO titer but followed the titer dynamics. Contrariwise, 39.3% (*n* = 22) did not treat and did not follow the titer, and only 8.9% (*n* = 5) of them treated asymptomatic patients with high ASLO titer.

Our last question was meant to identify the reasons for referring a patient with GAS-pharyngitis to a specialist, such as a pediatrician. Therefore, the most common reasons reported by our GPs sample were worsening of symptoms despite treatment (35.71%, *n* = 20), development of complications (e.g., acute glomerulonephritis) (26.78%, *n* = 15), exhaustion of outpatient treatment options (23.23%, *n* = 13), and low compliance to oral treatment (14.28%, *n* = 8). Women (w) asked for a specialist opinion especially if symptoms worsen despite treatment (w: 38.29%, *n* = 18/47 vs. m: 22.22%, *n* = 2/9), while men (m) mainly if complications occurred (m: 33.33%, *n* = 3/9 vs. w: 23.4%, *n* = 11/47), but with no statistically significant difference (*p* = 0.246 and *p* = 0.367, respectively).

## 4. Discussion

GAS-pharyngitis is an ancient disorder with major implications in pediatric population due to its great incidence, its associated complications and its recurrence pattern. A recent study performed in an Emergency Department from Romania concluded that the incidence of GAS-pharyngitis is high in children reaching almost 30% [14]. Our study raised important concerns regarding the management of this infection due to wide-variety of answers to each questions proving that there is no consensus on how to diagnose or when and how to treat GAS-pharyngitis in Romanian children. Nevertheless, we noticed that younger GPs tend to have a more appropriate approach regarding the diagnosis and treatment of this condition, following the international recommendations when compared to older GPs. Thus, not only did younger GPs used the Centor Criteria to diagnose the GAS-pharyngitis significantly more often (*p* = 0.027) but they also preferred to use targeted antibiotics for treating GAS-pharyngitis.

The Centor Criteria involves scoring the presence of different clinical signs/symptoms as it follows: absence of cough (1), temperature > 38 °C (1), swollen and tender anterior cervical nodes (1), swollen tonsils or with exudates (1), age between 3 and 14 years (1), between 15 and 44 years (0), and 45 years or above (−1). According to the American guidelines, those who fulfill a score of 1 or below carry a very low risk of having GAS-pharyngitis and they do not need further testing or antibiotics. Patients with a score of 2 or 3 definitely require an RADT or throat culture to confirm the infection, which if positive, requires antibiotics. In the setting of a score of 4 or above, the guidelines recommend the use of empirical antibiotics and if possible, RADT or throat culture before the antibiotics, which will be further guided by culture and antibiogram when possible [15]. Regarding the comparison of Centor and McIsaac scores, a recent study concluded that they have similar performance, and a score ≤0 certainly rules out GAS infection [16]. Nevertheless, it was underlined in a pediatric study that clinical picture is not reliable as a single test for confirming GAS-pharyngitis, and it should be confirmed by a laboratory test [17]. Also, clinical signs are not always objective since they could be misinterpreted in certain cases by the physician either being misled by the patient or due to his poor experience in a certain field as in our study where 10 of the GPs declared cough as a suggestive sign for GAS-pharyngitis. Clinical evaluation not only has a low sensitivity and specificity rate but it was proven that it has a positive predictive value of 44.9% and a negative one of 57.5%, leading to the risk of underdiagnosing GAS-pharyngitis, resulting in potential life-threatening complications [18]. Still, we must acknowledge that laboratory tests are not always available, especially in developing countries and, therefore, the value of the clinical criteria should not be underestimated. Moreover, certain risk factors were highlighted in increasing the prevalence of GAS-pharyngitis such as poor housing conditions, tobacco smoke, and indoor pollution [19]. These factors should be considered in association with clinical criteria for the diagnosis of GAS-pharyngitis in countries where other tests cannot be performed, especially since those countries usually have the highest rate of GAS-associated complications [19].

In terms of RADT, a recent review pointed out that this test has a sensitivity of 86% and specificity of 95% explaining that amongst 100 children with GAS-pharyngitis, 86 would be correctly diagnosed, and 16 would not receive antibiotic treatment due to the false negative test. At the same time, of 100 pediatric patients with non-GAS-pharyngitis, 95 would be properly classified, while 5 would receive unnecessary antibiotic treatment being falsely detected with GAS [7]. Culture from throat swabs remains the most reliable method to diagnose GAS-pharyngitis, but it is rather impractical in clinical practice due to its time delay in providing the results. Therefore, RADT is definitely a better option in clinical setting based on its relatively high sensitivity and its ability to render the results within minutes resulting in the most important outcome, i.e., to initiate antibiotic treatment as soon as possible for patients with GAS-pharyngitis [20,21,22,23]. Nevertheless, the most important issue with throat swabs is that they do not have the ability to differentiate between healthy GAS carriers and the disease resulting in a major clinical dilemma when to consider the symptoms to be causes by GAS or other pathogen, most often a viral one that would definitely not necessitate antibiotics [24]. GAS-carriage in healthy individuals might be a real problem in practice since it definitely challenges the clinical evaluation and management of patient complaining of sore throat that might in fact have a viral etiology despite the positive throat swab [24,25,26]. It was reported that prevalence of GAS carriage is higher in children and adolescents depending on other factors also such as population or season [25].

Our study proved that only 25% (*n* = 14) of the GPs used the Centor Criteria to diagnose GAS-pharyngitis, and even less, 10.7% (*n* = 6) of them requested RADT. Still, 42.9% (*n* = 24) of them requested throat culture for the diagnosis of GAS-pharyngitis. Similar findings were reported by Boyarchuk et al. indicating that only 20% of the Ukrainian pediatricians involved in their study used Centor or McIsaac criteria for diagnosing sore throat [27]. Contrariwise, Swedish GPs consider that solely clinical picture might be enough for typical cases of sore throat [28]. Still, the trends are constantly changing since a more recent study performed in Sweden revealed that 94% of the patients presenting with sore throat benefited by a RADT in primary health care [29]. Regarding RADT, a survey performed in Spain highlighted similar findings when compared to our study, indicating that only 12.7% of the GPs included in the study used this test in the management of patients with sore throat [30]. Germany seems to have the most appropriate approach in terms of using diagnostic tools in primary care practices according to the study of Peiter et al. who proved that 60% of the physicians use these tests in patients with sore throat, among which 25% recommend testing before prescribing antibiotics, 39% in patients with severe symptoms, 40% in patients refractory to treatment, and 25% in other situations [31].

The most important differential diagnoses mentioned by our participants were the viral causes. Taking into account that swollen tonsils associated with white exudates was the most common clinical sign reported in our study for patients with suspected GAS-pharyngitis, we noticed that if this sign was present, 19.64% (*n* = 11) of GPs used the Centor Criteria for the diagnosis, 7.14% (*n* = 4) used RADT and 39.28% (*n* = 22) required throat culture. Therefore, it would be useful to implement a standard diagnostic strategy among GPs from Romania and to explain the limitations and benefits of each diagnostic test in order to increase the use of RADT among Romanian children with suspected GAS-pharyngitis. Moreover, the combination between clinical score and a laboratory method would be the most appropriate way to differentiate between the carrier status and the acutely ill patient. This statement is also sustained by the current guidelines [32]. A recently described diagnostic tool consists of determining bacterial DNA from the saliva which was indicated by a recent study to have a sensitivity of 79% and a specificity of 91% [33].

According to the ESCMID Guidelines antibiotic treatment is indicated only in high-risk patients with positive RADT or other laboratory tests for preventing post-GAS complications [32]. The same experts stated that antibiotics are not necessary in patients mild symptoms (0–2 Centor score), while in those with more severe presentation (3–4 Centor score) antibiotics should be judiciously considered taking into account their negative impact on the gut microbiota, increased antibacterial resistance and costs [32]. Contrariwise, The World Health Organizations (WHO) Guidelines recommend antibiotic treatment for all children and adolescents with a sore throat who test positive for GAS in order to prevent rheumatic fever and rheumatic heart disease [2]. Penicillin V remains the first-choice treatment for GAS-pharyngitis, and its efficacy increases considerably if administered for 10 (long-course) [2,32]. The IDSA Guidelines mentioned both penicillin and amoxicillin as election drugs in non-allergic patients with GAS-pharyngitis due to their narrow activity spectrum, low risk of adverse reactions and modest costs Nevertheless, several debates were reported regarding the treatment option and duration taking into account that a long-term course of penicillin might have several adverse effects. Thus, several studies proposed shorter-courses of other antibiotics such as cephalosporins or macrolides [34]. A recent review compared the effectiveness of these treatments and concluded that short-course penicillin has a lower effectiveness in comparison to long-course penicillin V, no difference was found between short-course macrolides and long-course penicillin V, but a higher effectiveness was noticed for short-course cephalosporins versus long-course penicillin V [34]. Taking into account the increasing antimicrobial rates reported by different studies, the use of penicillin V for GAS-pharyngitis should remain the first option of treatment [35,36]. In our study, the most preferred antibiotics were amoxicillin-clavulanate and penicillin V, the older GPs preferring especially penicillin. More than half of the GPs included in the study prescribed a long-course of antibiotic (10 days or more) for the treatment of GAS-pharyngitis in children. Similarly, Spanish GPs were reported to prefer mainly amoxicillin (52.7%) and amoxicillin + clavulanate (31.2%) for treating sore throat, while penicillin V was used less frequently (11.9%) [30]. Unfortunately, the same study underlined that one in five GPs prescribed antibiotics even in patients with a possible viral infection [30]. Moreover, it seems that Swedish GPs guide their decision to use of antibiotics depending on the value of C-reactive protein, and not RADT, prescribing antibiotics in patient with increasing values of C-reactive protein and negative RADT [29]. Likewise, Danish GPs also rarely follow the guideline recommendations in children with sore throat [37]. The approach of German GPs regarding sustaining the use of testing in patients with sore throat proved that it might reduce the unjustified use of antibiotics lowering, therefore, the antimicrobial resistance. [31] A neighboring country of Romania, Bulgaria reported in a longitudinal study from 1998 to 2014 that more than 34% of the upper respiratory tract infections in Bulgarian children are caused by *Streptococcus pyogenes* [38]. A study published this year (2025) that included 82 Bulgarian children with either scarlet fever or GAS-pharyngitis highlighted the same preference of Bulgarian physicians like Romanian ones for amoxicillin-clavulanate regarding the treatment of GAS infection and sore throat [39]. The authors also pointed out that all strains of *Streptococcus pyogenes* isolated in their study were susceptible to penicillin reinforcing the statement that penicillin should be the first choice in treating children with GAS-pharyngitis. Hungary, another neighbor of Romania seems to follow the same approach regarding the GP’s preference for using amoxicillin-clavulanate in children with sore throat and *Streptococcus pyogenes* infection [40]. Similar overall susceptibility of *Streptococcus pyogenes* to penicillin was also noticed in Hungary [41]. In fact, a recent review highlighted three different strategies in managing pediatric GAS-pharyngitis worldwide: (1) the first group sustaining the use of antibiotics in all children diagnosed with GAS-pharyngitis for preventing acute rheumatic fever; (2) the second group considering that GAS-pharyngitis is a self-limited disorder, and antibiotics are recommended only in selected cases; (3) and the third group who tailor the prescription of antibiotics to the patient’s risk of acute rheumatic fever [36]

The limitations of this study consist mainly of the relatively small sample size, and the retrospective questionnaire-based type of the study that might involve recall bias. Nevertheless, this is the first study in our country to highlight the preferences of GPs in terms of pediatric GAS-pharyngitis, indicating the urgent need for implementing a national consensus for managing this condition in order to prevent a dramatic increase in antimicrobial resistance.

## 5. Conclusions

Although our study was limited to a region of Romania, it highlighted that in children suspected with GAS-pharyngitis, GPs initiate antibiotics treatment most commonly only based on clinical symptoms. Moreover, the GPs from the central area of Romania included in this study, stated that they prefer targeted antibiotic therapy, but they failed in using the appropriate diagnostic tools. Amoxicillin-clavulanate was the preferred antibiotic in short-course therapy. Our findings might represent a strong basis for further research on this topic involving other regions from Romania in order to eventually elaborate and implement a standard national protocol for diagnosing and treating children with GAS-pharyngitis based on the availability of diagnostic methods and antibiotics in our country. Moreover, it could lead to the implementation of national health strategies consisting in up-to-date GAS guidelines for GPs, as well as equipping family medicine practices with the appropriate diagnostic tools which could increase the accuracy of diagnosis and reduce unnecessary antibiotic use in children with GAS-pharyngitis.

## Figures and Tables

**Table 1:** Descriptive analysis of the evaluated variables.

Variables	Number (%)
*Your age? (years)*	*n* = 56
29–35	11 (19.6%)
36–45	22 (39.3%)
46–55	4 (7.1%)
56–65	12 (21.4%)
>65	7 (12.5%)
*Praxis in:*	*n* = 56
Urban area	34 (60.7%)
Rural area	22 (39.3%)
*Your gender*	*n* = 56
Male	9 (16.1%)
Female	47 (83.9%)
*Number of children treated in your practice*	*n* = 56
0–50	4 (7.1%)
51–100	4 (7.1%)
101–200	11 (19.6%)
>200	37 (66.1%)
*How many GAS-pharyngitis per month have you treated last year?*	*n* = 56
<5	18 (32.1%)
5–10	20 (35.7%)
>10	18 (32.1%)
*What symptoms you think are suggestive for GAS-pharyngitis?*	*n* = 56
Fever > 38 °C	48 (85.7%)
Swollen and tender anterior cervical adenopathy	46 (82.1%)
Swollen tonsils with white exudate	52 (92.9%)
Sore throat	41 (73.2%)
Lack of appetite	24 (42.9%)
Cough	10 (17.9%)
Vomiting	28 (50%)
*Do you use the CENTOR Criteria for diagnosis of GAS?*	*n* = 56
Yes	14 (25%)
No	42 (75%)
*Do you use a rapid antigen detection test (RADT) in your practice?*	*n* = 56
Yes	6 (10.7%)
No	50 (89.3%)
*Do you request throat culture in all cases when GAS-pharyngitis is suspected?*	*n* = 56
Yes	24 (42.9%)
No	32 (57.1%)
*You will make differential diagnosis with ….*	*n* = 56
Mononucleosis infectiosa	51 (91.1%)
Peritonsillar abscess	32 (57.1%)
Viral pharyngitis	45 (80.4%)
Scarlet fever	33 (58.9%)
Rhinopharyngitis	36 (64.3%)
*Do you start treatment after you have confirmed clinically the GAS-pharyngitis?*	*n* = 56
Yes, immediately	48 (85%)
Yes, but only after a positive throat swab result	8 (15%)
No	0 (0%)
*If YES, how you will treat the patient?*	*n* = 56
Targeted antibiotic therapy	39 (69.6%)
Empirical antibiotic therapy	21 (37.5%)
Symptomatic treatment	8 (14.3%)
Non-drug symptomatic treatment (home remedies)	4 (7.1%)
*How long will you treat the GAS patient?*	*n* = 56
5 days	2 (3.6%)
7 days	22 (39.3%)
10 days	31 (55.4%)
>10 days	1 (1.8%)
*Which antibiotic do you use most often as first choice?*	*n* = 56
Amoxicillin/clavulanic acid	31 (55.4%)
Penicillin V (po)	22 (39.3%)
Erythromycin	6 (10.7%)
Clindamycin	1 (1.8%)
3rd generation Cephalosporin	3 (5.4%)
2nd generation Cephalosporin	5 (8.9%)
Penicillin G (iv)	10 (17.9%)
*Does access to medicines influence antibiotic choice?*	*n* = 56
Yes	50 (89.3%)
No	6 (10.7%)
*Which route of antibiotic administration do you prefer?*	*n* = 56
Per oral	50 (89%)
Intravenous	5 (9%)
*Do you recall your patient for a follow-up visit?*	*n* = 56
Yes, always	54 (96.4%)
No	2 (3.6%)
Yes, only if its evolution is not appropriate	0 (0%)
*When do you refer the patient to a pediatric specialist?*	*n* = 56
From the onset	0 (0%)
If the symptoms worsen despite treatment	20 (35.71%)
If I have exhausted outpatient treatment options	13 (23.23%)
If the patient has a low compliance for p.o. treatment	8 (14.28%)
If complications appear (e.g., scarlet fever, acute glomerulonephritis, etc.)	15 (26.78%)
*Do you use the ASLO titer to follow up patients?*	*n* = 56
Yes	27 (48.2%)
No	29 (51.8%)
*Do you treat asymptomatic patients with high ASLO titers?*	*n* = 56
No	22 (39.3%)
No, but I follow the dynamics of ASLO values	29 (51.8%)
Yes	5 (8.9%)

## Data Availability

The data presented in this study are available on request from the corresponding author.

## Supplementary Materials

The following supporting information can be downloaded at: https://www.mdpi.com/article/10.3390/medicina61081408/s1, File S1: Group A streptococcus infection—Questionnaire for general practitioners.

## Abbreviations

The following abbreviations are used in this manuscript:

GAS	Beta-hemolytic group A streptococcus
GP	General practitioner
ENT	Ear–nose–throat
RADT	Rapid antigen detection test
