# Supplementary material for: General Practitioner’s Practice in Romanian Children with Streptococcal Pharyngitis
Source: Medicina (Kaunas). 2025 Aug 2;61(8):1408. doi: 10.3390/medicina61081408 (PMC12388020; doi:10.3390/medicina61081408)
Supplement: Supplementary file 1 [file medicina-61-01408-s001.zip › medicina-3756860-supplementary.pdf]

GROUP A STREPTOCOCCUS INFECTION - QUESTIONNAIRE FOR GENERAL PRACTITIONERS

Question 1: Your age? (years)
- a) 29-35
- b) 36-45
- c) 46-55
- d) 56-65
- e) >65

Question 2: *Your praxis is in:*
- a) Urban area
- b) Rural area

Question 3: Your gender:
- a) Male
- b) Female

Question 4: *Number of children treated in your practice:*
- a) 0-50
- b) 51-100
- c) 101-200
- d) >200

Question 5: *How many GAS-pharyngitis per month have you treated last year?*
- a) <5
- b) 5-10
- c) >10

Question 6: *What symptoms you think are suggestive for GAS-pharyngitis?*
- a) Fever >38°C
- b) Swollen and tender anterior cervical adenopathy
- c) Swollen tonsils with white exudate
- d) Sore throat
- e) Lack of appetite
- f) Cough
- g) Vomiting

Question 7: *Do you use the CENTOR Criteria for diagnosis of GAS?*
- a) Yes
- b) No

Question 8: *Do you use a rapid antigen detection test (RADT) in your practice?*
- a) Yes
- b) No

Question 9: *Do you request throat culture in all cases when GAS-pharyngitis is suspected?*

a) Yes
b) No

Question 10: *You will make differential diagnosis with ….*
a) Mononucleosis infectiosa
b) Peritonsillar abscess
c) Viral pharyngitis
d) Scarlet fever
e) Rhinopharyngitis

Question 11: *Do you start treatment after you have confirmed clinically the GAS-pharyngitis?*
a) Yes, immediately
b) Yes, but only after a positive throat swab result
c) No

Question 12: *If YES, how you will treat the patient?*
a) Targeted antibiotic therapy
b) Empirical antibiotic therapy
c) Symptomatic treatment
d) Non-drug symptomatic treatment (home remedies)

Question 13: *How long will you treat the GAS patient?*
a) 5 days
a) 7 days
b) 10 days
c) >10 days

Question 14: *Which antibiotic do you use most often as your first choice?*
a) Amoxicillin/clavulanic acid
a) Penicillin V (po)
b) Erythromycin
c) Clindamycin
d) 3rd generation Cephalosporin
e) 2nd generation Cephalosporin
f) Penicillin G (iv)

Question 15: *Does access to medicines influence your antibiotic choice?*
a) Yes
b) No

Question 16: *Which route of antibiotic administration do you prefer?*
a) Per oral
b) Intraveous

Question 17: *Do you recall your patient for a follow-up visit?*
a) Yes, always

b) No
c) Yes, only if its evolution is not appropriate

Question 18: *When do you refer the patient to a pediatric specialist?*

a) From the onset
b) If the symptoms worsen despite treatment
c) If I have exhausted outpatient treatment options
d) If the patient has a low compliance for p.o. treatment
e) If complications appear (e.g. scarlet fever, acute glomerulonephritis, etc.)

Question 19: *Do you use the ASLO titer to follow up patients?*

a) Yes
b) No

Question 20: *Do you treat asymptomatic patients with high ASLO titers?*

a) No
b) No, but I follow the dynamics of ASLO values
c) Yes
